# Square Dance the Key Factor of the Elevating Prevalence of Physical Activity in China

**Published:** 2019-10

**Authors:** Chao DENG, Ruibing FENG, Lihong KONG

**Affiliations:** College of Acupuncture and Orthopedics, Hubei University of Traditional Chinese Medicine, Wuhan, Hubei, 430065, China

## Dear Editor-in-Chief

Physical inactivity has become a global problem that may lead to substantial disease and economic burdens worldwide (1). What's worse, from 2001 to 2016, the problem did not improve. Overall, 96% of the world's population in 168 major countries, 27.5% adults were insufficient physical activity in 2016 worldwide, and even 37% happened in developed countries (2).

However, in East and Southeast Asia, there was a significant decline in this data. The paper points out that the reasons behind this phenomenon mainly come from China. In 2016, the prevalence of insufficient physical activity was only 14.1% in China which is much lower than the other major countries in the world. China has become one of the countries with the highest prevalence of physical activity in the world under the general trend of lack of activity in the world. In view of this phenomenon, the increased use of parks for the elderly in China is an important reason (2). Among all the park sports in China, square dancing is the fastest growing fitness sport in recent years, except walking. Square dancing is one of the most popular exercises for people over 50 in China, with participation reaching about 10%, according to data released by the State Sports Administration in 2015 (3). Therefore, the rise of square dance participation in recent years may be the real reason for the increasing prevalence of physical activity in China.

Square dance is a kind of mass fitness activity developed in China in recent years, which integrates fitness and dance with rhythmic music, and is carried out in public venues such as square, park and so on. It is called "square dance" because it is often seen in the square of Chinese cities (4). In recent years, with the increasing prevalence of square dance, it has also attracted the attention of researchers. Square dancing had a preventive and therapeutic effect on senile diseases such as cognitive disorder in the elderly (5). However, some of the negative factors caused by square dancing should also be aware, such as the music noise, occupation of public spaces, and so on (4). While vigorously promoting square dancing, some measures should be taken to reduce its impact on others, such as building more public green spaces in Chinese cities, inventing a new music delivery systems that could eliminate public music noise for group physical activities, and so on (4). Only by solving these problems can the square dance in China developed better and help to protect the health of the middle-aged and the elderly.
